# Evaluation of extravascular lung water and cardiac function by intrapartum bedside ultrasound: full-term preeclampsia vs. healthy controls

**DOI:** 10.3389/fcvm.2026.1709378

**Published:** 2026-02-12

**Authors:** Shi-jie Zhang, Shao-zheng He, Jing-jing Wu, Yong-jian Chen, Guo-rong Lyu

**Affiliations:** 1 Department of Ultrasound, Second Affiliated Hospital of Fujian Medical University, Quanzhou, Fujian, China; 2 Department of Obstetrics and Gynecology, Second Affiliated Hospital of Fujian Medical University, Quanzhou, Fujian, China; 3 Department of Clinical Medicine, Quanzhou Medical College, Quanzhou, Fujian, China

**Keywords:** echocardiography, hypertensive disorders of pregnancy, labor, lung ultrasound, pulmonary edema

## Abstract

**Background:**

Studies addressing full-term preeclampsia (PE) remain an area awaiting further exploration, and data concerning hemodynamic changes in women with PE during the initial 2 h postpartum remain elusive. This study aimed to observe the changes in extravascular lung water (EVLW), intravascular volume, and cardiac function in full-term PE before and after delivery and to investigate the differences in these parameters between PE patients and healthy women.

**Methods:**

This was a prospective, observational study. A total of 16 women were included in the PE group, and 32 were included in the control group. Bedside ultrasound examinations were performed 24 h before delivery, 2 h after delivery, and 24 h after delivery. A semiquantitative assessment of EVLW was conducted via the 28-rib interspace technique to calculate the echo comet score (ECS). The inferior vena cava collapsibility index (IVC-CI), left ventricular ejection fraction, left atrial volume index, left ventricular E/A ratio, e-peak deceleration time, left ventricular index of myocardial performance (LIMP), right ventricular fractional area change, right atrial volume index, right ventricular E/A ratio, and right ventricular index of myocardial performance (RIMP) were calculated.

**Results:**

ECS increased from 24 h before delivery to 2 h after delivery in both groups. From 2 to 24 h postpartum, only the control group presented a decrease in ECS. The ECS in the PE group remained consistently greater than that in the control group (all *P* < 0.001). The IVC-CI decreased in both groups from 24 h before delivery to 2 h after delivery and increased in both groups from 2 to 24 h postpartum, with the PE group showing a lower IVC-CI than that in the control group at 2 h postpartum (*P* = 0.048). The LIMPs in the PE group were greater than those in the control group at 24 h before delivery (*P* = 0.005), and the RIMPs in the PE group were greater than those in the control group at both 24 h before and 24 h after delivery (*P* < 0.001, *P* = 0.011).

**Conclusions:**

In this exploratory, hypothesis-generating study, EVLW and intravascular volume increased postpartum, with a greater increase observed in PE women, who also have a higher risk of pulmonary edema and a greater intravascular volume load. Intrapartum bedside ultrasound can be used for real-time monitoring of maternal EVLW, intravascular volume status, and cardiac function, aiding in the monitoring the condition and fluid management of PE patients.

## 1 Introduction

Preeclampsia (PE) is a severe complication of pregnancy, contributing to numerous cases of maternal, fetal, or neonatal mortality each year (1–3). Inappropriate fluid management can lead to insufficient organ perfusion or excessive lung water (4, 5). During labor, each uterine contraction causes approximately 300–500 mL of blood to enter the systemic circulation, and the excitation of the sympathetic nervous system results in increased blood pressure and heart rate, leading to a significant increase in cardiac output (6). Postpartum, the release of uteroplacental circulation and the effects of uterine involution cause additional blood flow into the circulation, further elevating cardiac output (6, 7). The maternal cardiovascular system experiences greater stress during and after delivery than before delivery, highlighting the importance of rapidly and accurately evaluating maternal hemodynamics (8).

Transthoracic echocardiography provides a rapid and non-invasive assessment of cardiac morphology and function (9–11). By measuring the inferior vena cava (IVC) and calculating the inferior vena cava collapsibility index (IVC-CI), transabdominal ultrasound can quickly and non-invasively evaluate intravascular volume (12, 13). When the subcostal view of the IVC is unclear, assessment can be performed through a transhepatic view (14, 15). Point-of-care lung ultrasound is useful for assessing maternal lungs and allows for a semiquantitative evaluation of extravascular lung water (EVLW), demonstrating good repeatability (16, 17).

A study indicated that at 1 day before and 1 day after delivery, PE patients (with a median gestational age of 33 + 0 weeks) had greater EVLW than that in healthy controls (18). Additionally, women with PE may exhibit myocardial dysfunction (8, 18). Gestational age is an important determinant of hemodynamics during pregnancy; however, no studies have reported changes in hemodynamics during labor in full-term PE patients. Furthermore, the first 2 h postpartum (the fourth stage of labor) is a critical period for monitoring changes in hemodynamics; however, data on hemodynamic changes in PE women during this postpartum period are lacking.

Therefore, this study aimed to observe changes in EVLW, IVC-CI, and cardiac function in full-term pregnant women with PE at 24 h before delivery, 2 h after delivery, and 24 h after delivery and to investigate differences in these parameters between PE patients and healthy controls. This study was designed as an exploratory, hypothesis-generating investigation.

## 2 Methods

### 2.1 Study population

This was a prospective, observational study. A total of 16 full-term PE patients who delivered at the obstetrics center of a tertiary hospital from August 2023 to October 2024 were enrolled. The diagnostic criteria for PE were based on national guidelines (19). PE was defined as hypertension (systolic blood pressure ≥140 mmHg and/or diastolic blood pressure ≥90 mmHg on two occasions at least 4 h apart) after 20 weeks of gestation, along with at least one of the following criteria: (1) proteinuria ≥0.3 g/24 h, protein/creatinine ratio ≥0.3, or random urine protein ≥(+) (when quantitative protein assessment is not feasible) or (2) in the absence of proteinuria, at least one organ or system being affected, including the heart, lungs, liver, kidneys, or hematological, digestive, and nervous systems, or evidence of placental–fetal effects. Severe PE was defined as the presence of any of the following: (1) uncontrolled elevated blood pressure (systolic pressure ≥160 mmHg and/or diastolic pressure ≥110 mmHg); (2) persistent headache, visual disturbances, or other central nervous system abnormalities; (3) persistent upper abdominal pain and subcapsular liver hematoma or liver rupture; (4) elevated alanine transaminase or aspartate transaminase; (5) renal insufficiency (proteinuria >2.0 g/24 h, urine output <400 mL/24 h or <17 mL/h, or serum creatinine >106 μmol/L); (6) hypoproteinemia with ascites, pleural effusion, or pericardial effusion; (7) persistent thrombocytopenia (platelet count <100 × 10^9^/L), microangiopathic hemolysis (anemia, elevated lactate dehydrogenase, or jaundice); (8) heart failure; (9) pulmonary edema; and (10) fetal growth restriction, oligohydramnios, fetal death, or placental abruption.

Additionally, 32 healthy controls, matched with the PE group by maternal and gestational age, were selected during the same period. Upon admission, each participant underwent an examination by an obstetrician or midwife to assess cervical dilation and the stage of labor. Women in the active stage of labor were not considered for inclusion. The inclusion criteria were singleton term pregnancy, maternal age not less than 18 years, and the ability to understand and provide informed consent. The exclusion criteria included any systemic diseases that could lead to pulmonary edema (such as heart disease, asthma, lung disease, immune disease, and diabetes), upper respiratory tract infection, poor image quality (due to factors such as obesity and breast thickening), postpartum complications affecting the pulmonary or circulatory system (e.g., postpartum hemorrhage), and addiction to tobacco, alcohol, or drugs.

### 2.2 Ultrasound procedure

All ultrasound examinations were performed by a researcher (S-jZ) who was trained in lung ultrasound and echocardiography. A Mindray Z6 ultrasound diagnostic system (Mindray Bio-Medical Electronics Co., Ltd., Shenzhen), equipped with a 3C5P abdominal probe and a 2P2P cardiac probe, with frequencies of 3.5–6.5 MHz and 2.0–5.0 MHz, respectively, was used. For longitudinal monitoring of changes in various indicators, measurements were taken 24 h before delivery, 2 h after delivery, and 24 h after delivery. All ultrasound examination results were saved for offline analysis. Two researchers (S-jZ and Y-jC) independently determined the number of B-lines via lung ultrasound. In cases of disagreement, a third researcher (G-rL) made the final decision. All the researchers had a minimum of 3 years of experience in lung ultrasound diagnostics.

A semiquantitative assessment of EVLW was conducted via the 28-rib interspace technique to calculate the echo comet score (ECS) (18). The 28-rib interspace technique involves scanning specific points in the midaxillary line, anterior axillary line, midclavicular line, and parasternal line at the left 2–4 and right 2–5 intercostal spaces and then calculating the number of B-lines at each point. The total count of B-lines across all points represents ECS. B-lines refer to high-echo lines originating from the pleura, extending laser-like to the screen edge without attenuation, and sliding with respiration (20). In the ECS, EVLW is classified as absent (ECS ≤5), mild (6–15), moderate (16–30) , or severe (≥31 or diffuse distribution ) (20).

Using transabdominal ultrasound, a longitudinal section of the IVC was displayed under the xiphoid or the costal margin in the supine position. Measurements of the end-expiratory inferior vena cava diameter (IVCe) and end-inspiratory inferior vena cava diameter (IVCi) were taken at the proximal end of three hepatic veins converging into the IVC, approximately 0.5–1.0 cm from the right atrium. The measurements were taken perpendicular to the long axis of the IVC. The calculation was performed via the formula IVC−CI=[(IVCe−IVCi)/IVCe]×100%.

The echocardiography examinations were conducted according to the guidelines (10, 11, 13). Measurements of the left ventricular ejection fraction (LVEF), left atrial volume index (LAVI), left ventricular E/A ratio (LV E/A), e-peak deceleration time (EDT), left ventricular index of myocardial performance (LIMP), right ventricular fractional area change (RVFAC), right atrial volume index (RAVI), right ventricular E/A ratio (RV E/A), and right ventricular index of myocardial performance (RIMP) were taken, with the heart rate measured before each examination.

### 2.3 Statistical analysis

Continuous variables are represented as medians and interquartile ranges, whereas categorical variables are represented as frequencies and percentages. Differences between the PE and control groups were analyzed via the Mann‒Whitney *U* test for continuous variables and the chi-square test or Fisher's exact test for categorical variables. The differences in the ultrasound evaluation results between 24 h before delivery, 2 h after delivery, and 24 h after delivery were analyzed via the Friedman test, followed by pairwise comparisons conducted via the Q test (PMCMRplus R package) and Holm correction. The Friedman test was used to account for within-subject correlations across repeated measurements over time. Owing to the limited sample size and the exploratory nature of this study, formal time–group interaction testing using mixed-effects models was not performed. Instead, temporal changes were assessed within each group, and between-group comparisons were conducted at each time point. Statistical analysis was conducted via R (Ver. 4.4.1; R Development Core Team, Austria, Vienna). *P* ≤ 0.05 was considered statistically significant. Owing to the prospective and exploratory nature of this study, there are currently no suitable reference indicators for sample size calculation.

### 2.4 Ethics statement

This study was approved by the Medical Research Ethics Committee of the Second Affiliated Hospital of Fujian Medical University (No. 2022278 and No. 2024531). All participants provided written informed consent before inclusion.

## 3 Results

### 3.1 Study population

The PE and control groups were comparable in terms of maternal age, body mass index, body surface area, nulliparous rate, gestational age, net fluid intake 2 h postpartum, and cesarean section rate (Table 1). In the PE group, all 16 patients had proteinuria, and 3 patients (18.75%) were diagnosed with severe PE due to a systolic blood pressure ≥160 mmHg and/or diastolic blood pressure ≥110 mmHg. Intrapartum variables potentially affecting intravascular volume were recorded. Oxytocin was administered to one patient in the PE group and seven patients in the control group. In the PE group, two patients underwent vaginal delivery without analgesia, and the remaining patients underwent cesarean delivery under spinal anesthesia. In the control group, nine patients had vaginal delivery, among whom five received epidural analgesia, while the remaining participants underwent cesarean delivery under spinal anesthesia. Owing to the small sample size and unbalanced distribution of these variables, they were not included in further statistical analysis.

**Table 1:** Clinical characteristics of 16 patients with preeclampsia and 32 healthy controls.

Characteristic	Preeclampsia	Control	*P*
Maternal age, year	30.50 (5.25)	30.00 (5.25)	0.317
Body mass index, kg/m^2^	29.32 (2.59)	27.29 (4.66)	0.054
Body surface area, m^2^	1.72 (0.08)	1.70 (0.19)	0.406
Nulliparous	9 (56.25%)	10 (31.25%)	0.175
Gestational age, week	38 + 4 (1 + 0)	38 + 4 (1 + 4)	0.710
Estimated net fluid intake^a^, mL	−375.00 (675.00)	−100.00 (462.50)	0.142
Vaginal delivery	2 (12.50%)	9 (28.12%)	0.395

Data were represented as median (interquartile range) or *n* (%).

^a^ Net fluid intake = estimated total intravenous fluid intake − estimated blood loss ×3.

### 3.2 ECS

**Figure 1:**
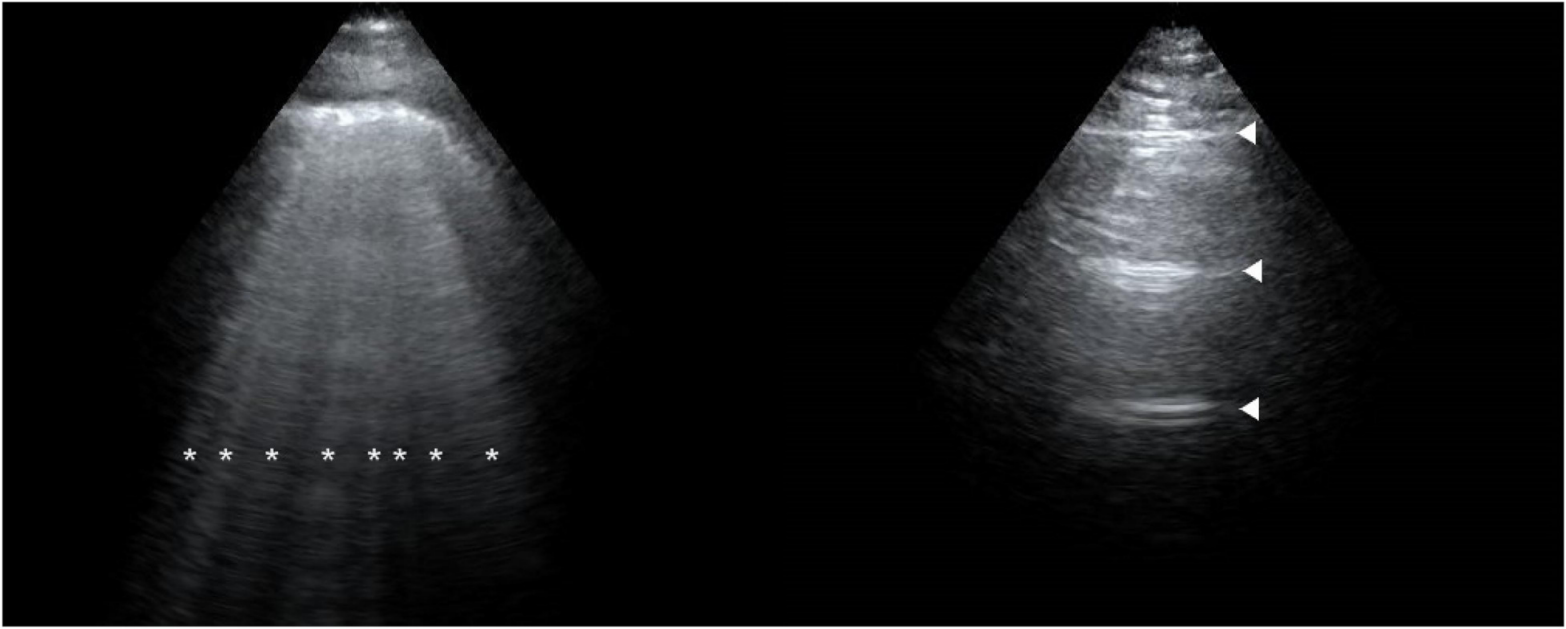
Lung ultrasound performance of a preeclampsia patient (left) and a healthy patient (right) at 2 h postpartum. The image of a preeclampsia patient is filled with B-lines (*). The image of a healthy patient is shown as A-lines (▴).

The ECS in the PE group was greater than that in the control group at 24 h before delivery, 2 h postpartum, and 24 h postpartum (all *P* < 0.001). With respect to the degree of EVLW increase, at 24 h before delivery, nine patients in the PE group presented mild increases, whereas none in the control group did. At 2 h postpartum, 11 patients in the PE group presented mild increases, and 1 patient presented moderate increases, whereas 5 patients in the control group presented mild increases. At 24 h postpartum, six patients in the PE group presented mild increases, two patients presented moderate increases, and one patient in the control group presented mild increases (Table 2). Figure 1 shows the lung ultrasound performance of PE patients and healthy controls at 2 h postpartum.

**Table 2:** Differences in lung ultrasound between the preeclampsia and control groups at different time points.

Time points Index	24 h before delivery		*P*	2 h after delivery		*P*	24 h after delivery		*P*
	Preeclampsia	Control		Preeclampsia	Control		Preeclampsia	Control	
ECS	6.00 (4.25)	2.00 (1.00)	<0.001*	7.50 (6.25)	3.00 (2.25)	<0.001*	5.50 (8.75)	1.00 (2.00)	<0.001*
EVLW									
None	7 (43.75)	32 (100.00)	<0.001*	4 (25.00)	27 (84.38)	<0.001*	8 (50.00)	31 (96.88)	<0.001*
Mild	9 (56.25)	0 (0)	<0.001*	11 (68.75)	5 (15.62)	<0.001*	6 (37.50)	1 (3.12)	<0.001*
Moderate	0 (0)	0 (0)	–	1 (6.25)	0 (0)	<0.001*	2 (12.50)	0 (0)	<0.001*^·^
Severe	0 (0)	0 (0)	–	0 (0)	0 (0)	–	0 (0)	0 (0)	–

Values reported as median (interquartile range) or frequency (%) as appropriate.

ECS, echo comet score; EVLW, extravascular lung water.

* Means statistically significant.

**Figure 2:**
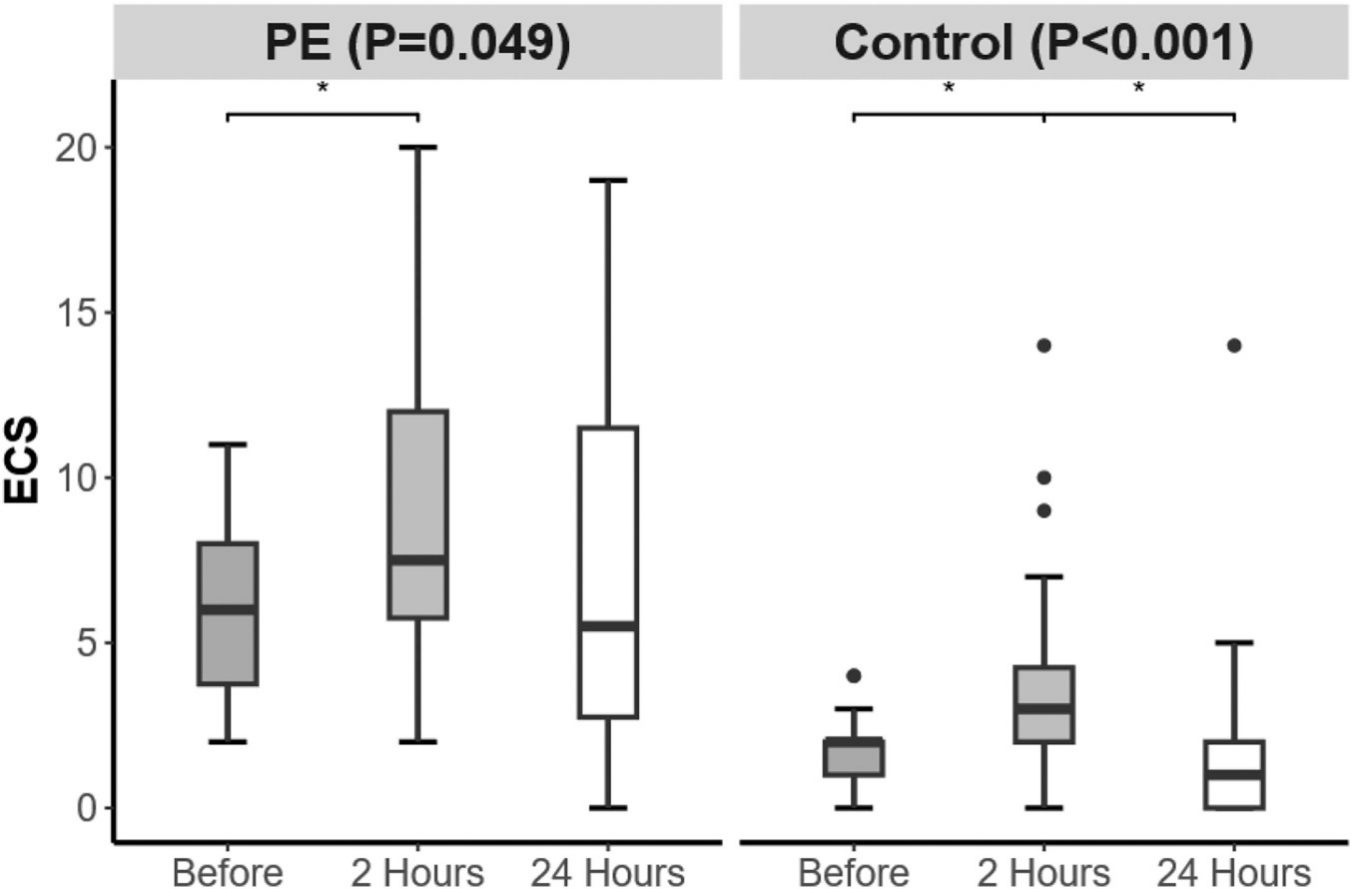
Changes in echo comet score (ECS) over time in the preeclampsia and control groups. Significant difference is indicated by *.

From 24 h before delivery to 2 h postpartum, the ECS in both groups increased. From 2 to 24 h postpartum, only the control group presented a decrease in ECS (Figure 2).

### 3.3 IVC-CI

**Figure 3:**
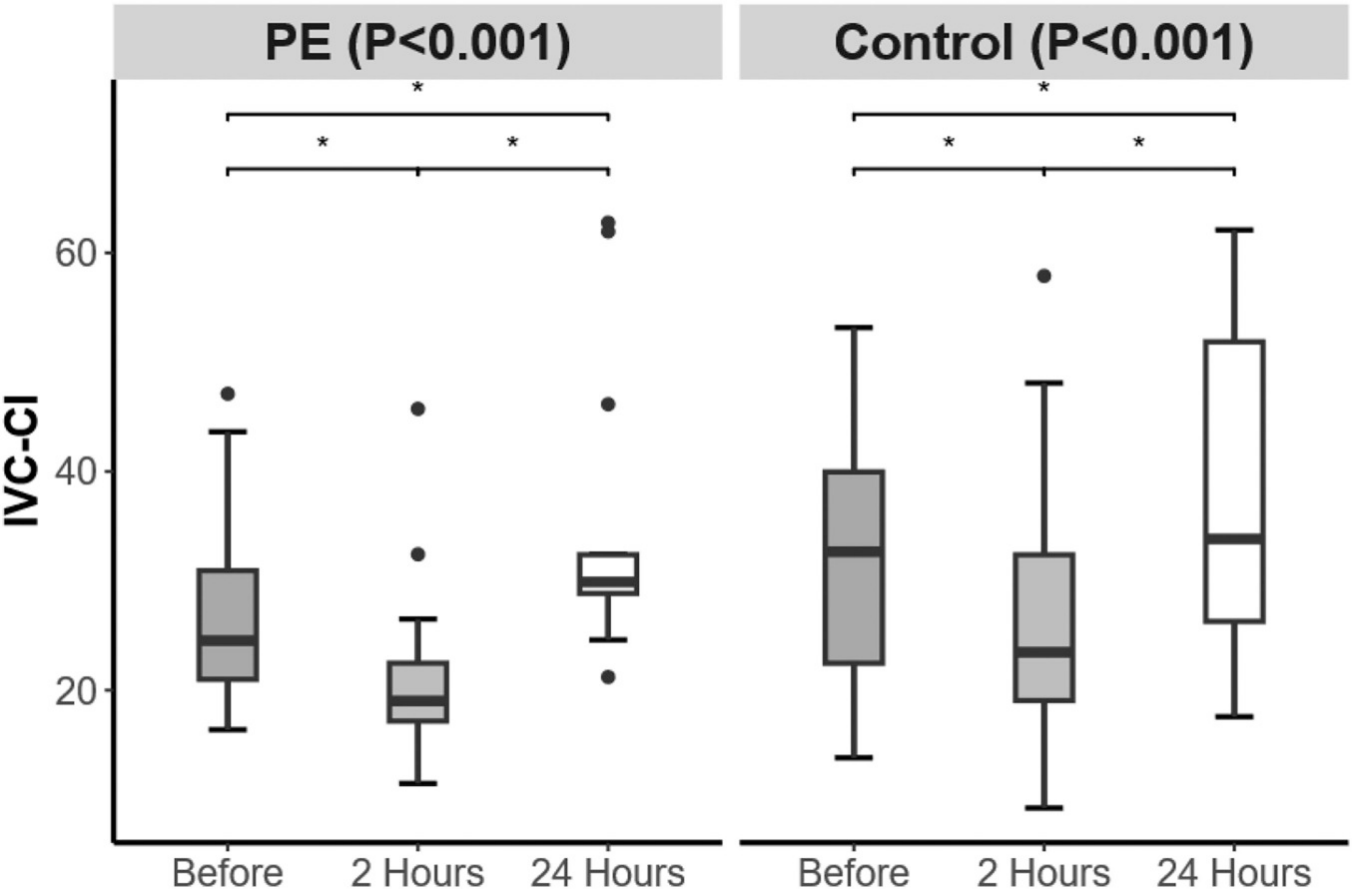
Changes in inferior vena cava collapsibility index (IVC-CI) over time in the preeclampsia and control groups. Significant difference is indicated by *.

The IVC-CI in the PE group was lower than that in the control group at 2 h postpartum (*P* = 0.048) (Table 3). From 24 h before delivery to 2 h after delivery, both groups presented a decrease in the IVC-CI. From 2 to 24 h postpartum, both groups presented an increase in IVC-CI, which was greater than the level at 24 h before delivery (Figure 3).

**Table 3:** Differences of IVC-CI between the preeclampsia and control groups at different time points.

Time points Index	24 h before delivery		*P*	2 h after delivery		*P*	24 h after delivery		*P*
	Preeclampsia	Control		Preeclampsia	Control		Preeclampsia	Control	
IVCe, cm	1.10 (0.15)	1.07 (0.30)	0.425	1.37 (0.26)	1.41 (0.49)	0.793	1.43 (0.22)	1.33 (0.38)	0.425
IVCi, cm	0.81 (0.19)	0.71 (0.36)	0.120	1.11 (0.23)	1.07 (0.42)	0.623	1.01 (0.15)	0.87 (0.37)	0.158
IVC-CI, %	24.54 (9.94)	32.67 (17.50)	0.134	19.03 (5.30)	23.48 (13.33)	0.048*	29.90 (3.53)	33.84 (25.58)	0.279

Values reported as median (interquartile range).

IVCe, end-expiratory inferior vena cava diameter; IVCi, end-inspiratory inferior vena cava diameter; IVC-CI, inferior vena cava collapsibility index.

* Means statistically significant.

### 3.4 Left cardiac function

**Figure 4:**
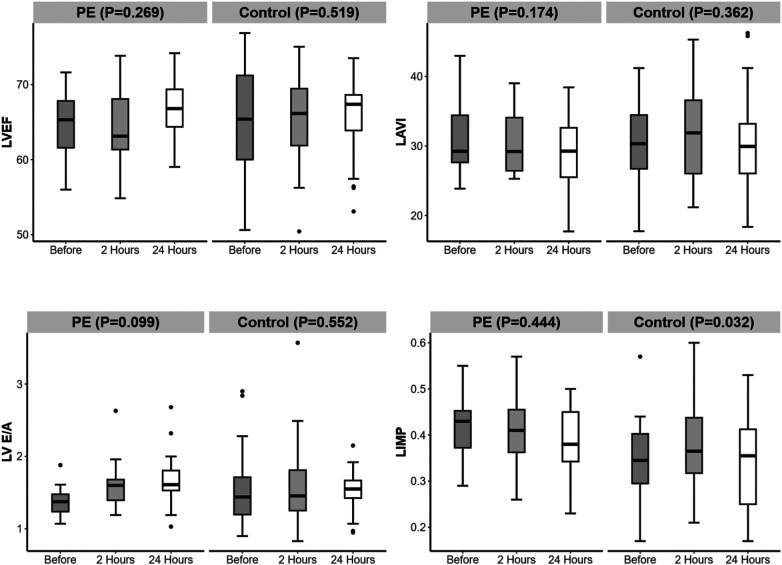
Changes in left heart function over time in the preeclampsia and control groups.

Among the left cardiac function indicators, the LIMP in the PE group was greater than that in the control group 24 h before delivery (*P* = 0.005) (Table 4). From 24 h before delivery to 24 h after delivery, there were no significant changes in LVEF, LAVI, LV E/A, EDT, or LIMP in either group (Figure 4).

**Table 4:** Differences in cardiac function between the preeclampsia and control groups at different time points.

Time points Index	24 h before delivery		*P*	2 h after delivery		*P*	24 h after delivery		*P*
	Preeclampsia	Control		Preeclampsia	Control		Preeclampsia	Control	
Left heart									
LVEF, %	65.31 (6.24)	65.40 (11.20)	0.686	63.13 (6.74)	66.15 (7.58)	0.400	66.81 (5.00)	67.38 (4.73)	0.853
LAVI, mL/m^2^	29.23 (6.77)	30.32 (7.74)	0.956	29.20 (7.63)	31.88 (10.55)	0.400	29.25 (7.11)	29.93 (7.14)	0.477
LV E/A	1.38 (0.24)	1.44 (0.52)	0.548	1.60 (0.29)	1.46 (0.56)	0.638	1.61 (0.28)	1.55 (0.24)	0.193
EDT, ms	177.00 (73.50)	186.00 (42.00)	0.809	201.00 (31.50)	201.00 (40.50)	0.913	192.00 (37.50)	201.00 (31.50)	0.482
LIMP	0.43 (0.08)	0.35 (0.11)	0.005*	0.41 (0.09)	0.37 (0.12)	0.255	0.38 (0.11)	0.36 (0.16)	0.139
Right heart									
RVFAC, %	50.13 (9.03)	47.34 (10.36)	0.123	51.55 (6.14)	50.55 (6.66)	0.269	48.11 (9.04)	46.59 (10.51)	0.242
RAVI, mL/m^2^	25.81 (7.59)	23.24 (8.98)	0.853	24.47 (6.69)	26.00 (9.87)	0.158	23.76 (5.62)	23.60 (9.77)	0.686
RV E/A	1.18 (0.33)	1.16 (0.31)	0.279	1.26 (0.30)	1.20 (0.31)	0.930	1.24 (0.33)	1.24 (0.22)	0.768
RIMP	0.40 (0.07)	0.28 (0.10)	<0.001*	0.38 (0.10)	0.38 (0.09)	0.562	0.38 (0.15)	0.29 (0.15)	0.011*

Values reported as median (interquartile range).

LVEF, left ventricular ejection fraction; LAVI, left atrial volume index; LV E/A, left ventricular E/A ratio; EDT, e-peak deceleration time; LIMP, left ventricular index of myocardial performance; RVFAC, right ventricular fractional area change; RAVI, right atrial volume index; RV E/A, right ventricular E/A ratio; RIMP, right ventricular index of myocardial performance.

* Means statistically significant.

### 3.5 Right cardiac function

**Figure 5:**
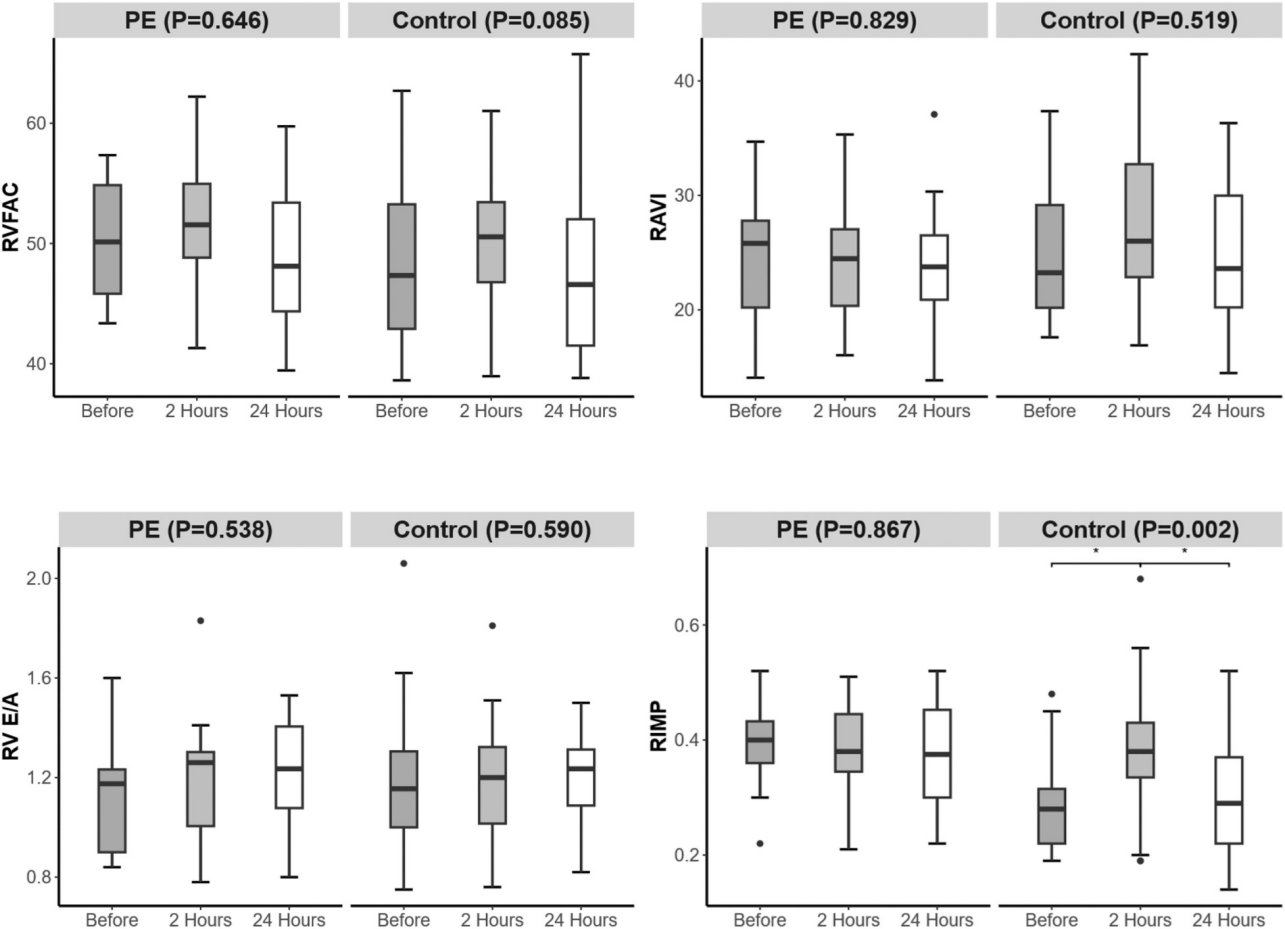
Changes in right heart function over time in the preeclampsia and control groups. Significant difference is indicated by *.

Among the right cardiac function indicators, the RIMP in the PE group was greater than that in the control group 24 h before and after delivery (*P* < 0.001, *P* = 0.011) (Table 4). From 24 h before delivery to 2 h after delivery, the RIMP in the control group increased. From 2 to 24 h postpartum, the RIMP in the control group decreased (Figure 5).

## 4 Discussion

In this study, we found that compared with full-term healthy women, full-term PE women had greater EVLW 24 h before delivery, 2 h after delivery, and 24 h after delivery and were more likely to develop pulmonary edema. This finding is consistent with the theory that PE patients are prone to pulmonary edema (1, 21). From 24 h before delivery to 2 h after delivery, the ECS in both groups increased, indicating a gradual accumulation of EVLW and an increasing likelihood of pulmonary edema. At 24 h postpartum, only the control group presented a decrease in ECS, indicating that healthy parturients have reduced EVLW through compensation by their cardiovascular system and that the likelihood of developing pulmonary edema decreases. In contrast, full-term PE parturients still have a higher risk of developing pulmonary edema due to insufficient compensatory ability caused by activation of pulmonary endothelial cells, activation of neutrophils, and decreased plasma colloid osmotic pressure (1). In the study by Ambrozic et al. (18), the median ECS 1 day before and 1 day after delivery was approximately 20, which was greater than that in our study (median ECS 5.5–7.5). Although the absolute ECS values in our study were lower, ECS should be interpreted as a semiquantitative indicator, in which dynamic changes and relative differences are more clinically informative than absolute thresholds, particularly in predominantly full-term, non-severe PE populations. The main reasons for this difference may include the following: (1) All patients in the study by Ambrozic et al. had severe PE, whereas only three patients (18.75%) in our study were ultimately diagnosed with severe PE; (2) the median gestational age in their study was 33 + 0 weeks, whereas 38 + 4 were diagnosed in our study. Premature birth may also contribute to the increase in EVLW (21). On the 4th day postpartum, the ECS in severe PE women declined to levels comparable to those in healthy parturients (18). Therefore, within 2–24 h postpartum, due to a further increase in EVLW, PE women are at increased risk of developing pulmonary edema, highlighting the need for focused monitoring during this period. These findings suggest an increased susceptibility to subclinical pulmonary edema and volume overload during the early postpartum period, highlighting the value of ECS for clinical monitoring rather than definitive diagnosis. Clinically, our findings suggest that the period from 2 to 24 h postpartum may represent a vulnerable window for pulmonary congestion in women with PE. During this interval, persistently elevated ECS without an early postpartum decline may indicate sustained extravascular lung water accumulation rather than transient physiological changes. These results support closer respiratory and hemodynamic monitoring during early postpartum care in PE patients, particularly with respect to fluid balance and early signs of pulmonary edema. Bedside lung ultrasound may serve as a practical, non-invasive tool to guide individualized fluid management and risk stratification during this high-risk period.

The peripartum increase in EVLW is likely multifactorial and may be related to physiologic autotransfusion from uterine contraction and placental separation, mobilization of extravascular fluid, and an acute increase in venous return around delivery (22). In women with preeclampsia, endothelial dysfunction, increased capillary permeability, and reduced plasma oncotic pressure may further impair fluid redistribution and contribute to greater EVLW accumulation (21). Notably, net fluid intake within 2 h postpartum did not differ between groups in our study, suggesting that the observed EVLW increase was unlikely to be primarily driven by intrapartum fluid administration.

IVC-CI can quickly, non-invasively, and objectively evaluate the intravascular volume of pregnant women and can be used for obstetric monitoring and management, such as postpartum hemorrhage (12, 23). Stawicki et al. (24) reported a negative correlation between the IVC-CI and central venous pressure (CVP), with a 3.3% decrease in the IVC-CI for every 1 mmHg increase in the CVP. An IVC-CI <25% may indicate high intravascular volume. Our study revealed that at 2 h postpartum, the IVC-CI in the PE group was lower than that in the control group. This may suggest a higher intravascular volume status and increased CVP in PE women at 2 h postpartum. From 24 h before delivery to 24 h postpartum, both groups showed a trend toward an initial decrease followed by an increase in IVC-CI, indicating that intravascular volume and CVP increased further postpartum than they did before delivery, followed by a gradual recovery as maternal compensation adapted. The IVC-CI is greater than 50% in normal adults (13). The median IVC-CI in our study ranged from 19.03% to 33.84%, which was significantly lower than the normal value. This difference may be partly explained by physiologic changes during late pregnancy and the peripartum period, including increased intravascular volume, elevated central venous pressure, and mechanical compression of the inferior vena cava by the gravid uterus (6, 25). In addition, IVC-CI values are known to be influenced by measurement conditions. In this study, IVC measurements were performed in the supine position under spontaneous respiration, with maximal and minimal diameters obtained during normal respiratory cycles according to standardized protocols. Therefore, the relatively low IVC-CI values observed likely reflect pregnancy-related hemodynamic adaptations rather than measurement-related bias. Notably, normal adult reference ranges may not be directly applicable to pregnant women, particularly during the peripartum period. These findings are consistent with the theory that intravascular volume increases during pregnancy and further increases postpartum (6, 7). To date, no studies have used the IVC-CI to explore changes in intravascular volume during delivery in full-term PE patients. The findings of our study indicate that within 2 h postpartum, the intravascular volume further increases, which is more pronounced in women with PE. IVC-CI could be utilized for real-time monitoring of maternal intravascular volume status, assisting in fluid therapy for PE patients.

Our study revealed that the LIMP of the PE group was greater than that of the control group at 24 h before delivery, and the RIMP was greater than that of the control group at 24 h before and after delivery. The index of myocardial performance (IMP) is the sum of the isovolumic contraction time (IVCT) and isovolumic relaxation time (IVRT), divided by the ejection time (ET), and is commonly used to evaluate overall cardiac function (10, 22, 26). The formula is expressed as IMP = (IVCT + IVRT)/ET. Possible reasons for the greater IMP in the PE group include the following: (1) prolonged IVRT; (2) increased afterload resulting in prolonged IVCT and shortened ET; and (3) increased afterload causing atrioventricular valve regurgitation, leading to shortened ET. This increase in IMP should be interpreted as reflecting altered myocardial performance under changing loading conditions, rather than definitive cardiac dysfunction. In our study, the RIMPs in the control group first increased but then decreased from 24 h before delivery to 24 h after delivery, with the highest level recorded at 2 h after delivery. The increase in RIMPs in the control group at 2 h postpartum may be related to the increased right ventricular volume load causing tricuspid regurgitation, leading to a shortened ejection time. Currently, no studies have used IMP to explore the cardiac function of PE patients before and after delivery. The results of our study indicate that IMP can be used to monitor the myocardial performance of postpartum women in real time, which can be used to observe the condition of PE patients.

Importantly, IMP is a composite index reflecting global myocardial performance rather than an isolated measure of systolic or diastolic function (10, 22). By integrating IVCT, IVRT, and ET, both LIMP and RIMP are sensitive to combined changes in ventricular relaxation, contractility, and loading conditions (22, 26). Although tissue Doppler-derived indices such as E/e′ ratio are more specific for estimating left ventricular filling pressure, IMP has been shown to be relatively less dependent on preload and heart rate and to have good reproducibility, particularly when tissue Doppler imaging is unavailable (26, 27). Therefore, in the present study, LIMP and RIMP were interpreted as indicators of overall myocardial performance rather than direct surrogates of conventional diastolic parameters, which is particularly relevant in the peripartum period characterized by rapid hemodynamic shifts (6).

Notably, a previous study (8) revealed that the severe PE group (average gestational age 32 + 5 weeks) had a greater left atrial volume, lower LV E/A, and longer EDT than those in the control group (average gestational age 38 + 3 weeks), which may indicate altered myocardial performance. However, in our study, no differences in the above indicators were detected between the PE and control groups. The possible main reason is that our study included full-term PE patients, and only three patients (18.75%) were ultimately diagnosed with severe preeclampsia due to a systolic blood pressure ≥160 mmHg and/or diastolic blood pressure ≥110 mmHg during hospitalization. Their cardiac function may be less affected, so their performance in terms of these indicators may be similar to that of normal healthy parturients. Future research may explore the above indicators before full-term pregnancy (such as 32 weeks) and compare their differences between the groups requiring early termination of pregnancy and those delivering at term after follow-up. These indicators may serve as potential references for determining whether to continue the pregnancy.

This study has several limitations. First, owing to the inclusion criteria for PE during full-term delivery and the inability to collect cases of nighttime delivery, the study's sample size was relatively small. In addition, only a small proportion of patients had severe preeclampsia, which may limit the generalizability of our findings to populations with more advanced disease and may partly explain the lower EVLW values compared with studies focusing exclusively on severe or early-onset PE. Given the relatively small sample size, these findings should be interpreted as hypothesis-generating rather than definitive. Second, owing to the lack of ultrasound equipment with tissue Doppler function, it is not possible to evaluate left ventricular diastolic function by measuring the E/e′ ratio. The absence of tissue Doppler and strain imaging limits the mechanistic interpretation of the observed changes in myocardial performance. However, the results of this study suggest that LIMP and RIMP may be other options in resource-limited places to assess cardiac function in clinical settings without tissue Doppler cardiac probes. Future studies could aim to validate and extend our findings through multicenter designs with larger sample sizes to improve generalizability. Inclusion of women at earlier gestational ages, particularly those with early-onset or severe PE requiring preterm delivery, may help clarify the trajectory of cardiopulmonary changes across disease severity. In addition, incorporation of advanced echocardiographic techniques, such as tissue Doppler imaging or speckle-tracking echocardiography, would allow more comprehensive assessment of ventricular function and provide external validation for the use of ultrasound-based indices in peripartum PE.

## 5 Conclusions

In this exploratory, hypothesis-generating study, we observed that both full-term PE parturients and normal healthy parturients experience increases in EVLW and intravascular volume postpartum, with a more pronounced increase in PE parturients, which may indicate a higher risk of pulmonary edema and a greater intravascular volume load. Bedside ultrasound during delivery can be used for real-time monitoring of EVLW, intravascular volume status, and cardiac function, which can be helpful not only in observing the condition of PE patients but also in fluid management.

## Data Availability

The raw data supporting the conclusions of this article will be made available by the authors, without undue reservation.
